# Passive surveillance of human African trypanosomiasis in Côte d’Ivoire: Understanding prevalence, clinical symptoms and signs, and diagnostic test characteristics

**DOI:** 10.1371/journal.pntd.0009656

**Published:** 2021-08-30

**Authors:** Minayégninrin Koné, Dramane Kaba, Jacques Kaboré, Lian Francesca Thomas, Laura Cristina Falzon, Mathurin Koffi, Cyrille Mambo Kouamé, Bernardin Ahouty, Charlie Franck Alfred Compaoré, Emmanuel Kouassi N’Gouan, Philippe Solano, Eric Fèvre, Philippe Büscher, Veerle Lejon, Vincent Jamonneau

**Affiliations:** 1 Unité de Recherche « Trypanosomoses », Institut Pierre Richet, Bouaké, Côte d’Ivoire; 2 Laboratoire de Biodiversité et Gestion des Ecosystèmes Tropicaux, Unité de Recherche en Génétique et Epidémiologie Moléculaire, UFR Environnement, Université Jean Lorougnon Guédé, Daloa, Côte d’Ivoire; 3 Unité de Recherche sur les Maladies à Vecteurs et Biodiversité, Centre International de Recherche-Développement sur l’Elevage en Zone Subhumide, Bobo-Dioulasso, Burkina Faso; 4 Unité de Formation et de Recherche en Sciences et Techniques, Université Nazi Boni, Bobo-Dioulasso, Burkina-Faso; 5 International Livestock Research Institute, Nairobi, Kenya; 6 Institute of Infection, Veterinary and Ecological Sciences, University of Liverpool, Liverpool, United Kingdom; 7 Projet de Recherches Cliniques sur la Trypanosomiase, Daloa, Côte d’Ivoire; 8 Unité Mixte de Recherche IRD-CIRAD 177, INTERTRYP, Institut de Recherche pour le Développement, Université de Montpellier, Montpellier, France; 9 Department of Biomedical Sciences, Institute of Tropical Medicine, Antwerp, Belgium; KARI-Trypanosomiasis Res Centre, KENYA

## Abstract

**Background:**

Little is known about the diagnostic performance of rapid diagnostic tests (RDTs) for passive screening of human African trypanosomiasis (HAT) in Côte d’Ivoire. We determined HAT prevalence among clinical suspects, identified clinical symptoms and signs associated with HAT RDT positivity, and assessed the diagnostic tests’ specificity, positive predictive value and agreement.

**Methods:**

Clinical suspects were screened with SD Bioline HAT, HAT Sero-*K*-Set and rHAT Sero-Strip. Seropositives were parasitologically examined, and their dried blood spots tested in trypanolysis, ELISA/*Tbg*, m18S-qPCR and LAMP. The HAT prevalence in the study population was calculated based on RDT positivity followed by parasitological confirmation. The association between clinical symptoms and signs and RDT positivity was determined using multivariable logistic regression. The tests’ Positive Predictive Value (PPV), specificity and agreement were determined.

**Results:**

Over 29 months, 3433 clinical suspects were tested. The RDT positivity rate was 2.83%, HAT prevalence 0.06%. Individuals with sleep disturbances (*p*<0.001), motor disorders (*p* = 0.002), convulsions (*p* = 0.02), severe weight loss (*p* = 0.02) or psychiatric problems (*p* = 0.04) had an increased odds (odds ratios 1.7–4.6) of being HAT RDT seropositive. Specificities ranged between 97.8%-99.6% for individual RDTs, and 93.3–98.9% for subsequent tests on dried blood spots. The PPV of the individual RDTs was below 14.3% (CI 2–43), increased to 33.3% (CI 4–78) for serial RDT combinations, and reached 67% for LAMP and ELISA/*Tbg* on RDT positives. Agreement between diagnostic tests was poor to moderate (Kappa ≤ 0.60), except for LAMP and ELISA/*Tbg* (Kappa = 0.66).

**Conclusion:**

Identification of five key clinical symptoms and signs may simplify referral for HAT RDT screening. The results confirm the appropriateness of the diagnostic algorithm presently applied, with screening by SD Bioline HAT or HAT Sero-*K*-Set, supplemented with trypanolysis. ELISA/*Tbg* could replace trypanolysis and is simpler to perform.

**Trial registration:**

ClinicalTrials.gov NCT03356665.

## Author summary

As human African trypanosomiasis (HAT) or sleeping sickness is approaching elimination, case management is progressively transferred from specialized teams to front line health care centres. This approach raises practical questions. What clinical symptoms and signs should trigger HAT testing? What rapid diagnostic tests (RDT) are suitable for screening? Which unconfirmed serological suspects should be examined further? During this study conducted in Côte d’Ivoire, individuals with sleep disturbances, motor disorders, convulsions, severe weight loss, or psychiatric problems were more often positive in RDTs. These symptoms and signs should trigger referral for HAT screening. Our results confirm appropriateness of the existing HAT screening strategy with SD Bioline HAT or HAT Sero-*K*-Set having specificities of 97.8% and 98.9%. Subsequent tests on dried blood spots from RDT positives were 93.3% to 98.9% specific, and increased the positive predictive value from below 15% up to 67%. For selection of RDT seropositives for additional parasitological examinations, trypanolysis on dried blood spots is suitable, but could be replaced by ELISA, which can be performed locally. The optimal diagnostic test algorithm for Côte d’Ivoire, in terms of cost-effectiveness, remains to be determined.

## Introduction

Sleeping sickness, or human African trypanosomiasis (HAT), is a parasitic disease that is fatal if left untreated. This disease is caused by an extracellular protozoan of the *Trypanosoma brucei* (*Tb*) species, which is transmitted by the tsetse fly. Two subspecies are pathogenic to humans: *Tb gambiense* (*Tbg*) and *Tb rhodesiense* (*Tbr*), causing distinct disease entities. The chronic HAT form, responsible for 98% of cases and endemic across Central and West Africa, is caused by *Tbg* [1,2]. HAT experienced an emergence / re-emergence phase with a peak number of 37,385 cases reported in 1998 [3]. Thanks to a very effective response, the number of cases decreased gradually, and HAT was included in the 2012 WHO roadmap on Neglected Tropical Diseases (NTDs), with the goal of elimination as a public health problem by 2020 [4]. With less than 1,000 cases reported since 2018, and an annual reduction of more than 40,000 km^2^ in the area reporting more than 1 case / 10,000 peoples / year, this target could be considered to have been achieved [5]. In the new WHO NTD roadmap, *Tbg* HAT is targeted for interruption of transmission by 2030 [6].

Active case finding, carried out by mobile teams, has largely contributed to HAT reduction but is no longer cost effective in low prevalence contexts [7]. The integration of HAT diagnosis into the routine activities of peripheral health centers, or passive screening, becomes crucial but requires diagnostic tests and algorithms adapted to the limited resources of these centers [1]. Indeed, the Card Agglutination Test for Trypanosomiasis (CATT) [8] used by mobile teams for population screening is not suitable for passive case detection: it is manufactured in multiple dose vials which expire quickly once opened, the preservation of CATT requires a cold chain, and a 12 Volt rotator is needed for performing the test. Specialized equipment and electricity are often not available in peripheral health centers, while the number of persons presenting to be tested for HAT can be low and vials of antigen may therefore not be used before expiry [9,10].

The advent of rapid diagnostic tests (RDTs) for individual HAT serodiagnosis, that are stable at ambient temperature and can be performed without additional equipment [11–14], is an important step towards integrating HAT surveillance in fixed health care facilities [15]. Several immunological tests, such as trypanolysis [16] and Enzyme Linked ImmunoSorbent Assay for *Tbg* (ELISA/*Tbg*) [17–19], and molecular tests, such as Loopamp *Trypanosoma brucei* Detection Kit (LAMP) [20,21,22] and real-time qPCR (RT-qPCR) [23–25], have also been developed for HAT diagnosis. While these serological and molecular assays are usually restricted to reference laboratories, they may still play a role in detecting the infection in pre-screened individuals [18,26].

Currently, little is known about the performance of diagnostic algorithms using RDTs in combination with additional serological and molecular tests for passive detection of suspected HAT cases requiring parasitological diagnosis. New, less toxic, oral drugs, efficient for all HAT stages [27,28] may offer opportunities for widened treatment of HAT suspects identified by RDT and/or other serological and molecular tests. Empirical data on the performance of different diagnostic algorithms are therefore needed to inform HAT surveillance, innovative case management and elimination efforts. The objectives of the present study were i) to determine the apparent prevalence of HAT in endemic foci of Côte d’Ivoire under passive surveillance; ii) to identify clinical symptoms and signs associated with RDT positivity and; iii) to assess the specificity, Positive Predictive Value (PPV) and agreement between the diagnostic tests used.

## Methods

### Ethics statement

This study corresponds to the Ivorian part of the DiTECT-HAT-WP2 multi-country diagnostic clinical trial (Diagnostic Tools for Human African Trypanosomiasis Elimination and Clinical Trials, work package 2, passive case detection, https://www.ditect-hat.eu/), which, before its implementation in Côte d’Ivoire received ethical clearance from the Advisory Committee on Deontology and Ethics (plenary meeting of 17–20 October 2016) of the French National Institute for Research on Sustainable Development (IRD), of the Institutional Review Board of the Institute of Tropical Medicine in Antwerp Belgium (reference 1133/16), of the Ethics Committee of the University of Antwerp (Belgian registration number B300201730927) and of the National Research Ethics Committee, Ministry of Public Health and Hygiene in Côte d’Ivoire (reference 076 // MSHP / CNER-kp). The DiTECT-HAT-WP2 project is registered on ClinicalTrials.gov, ID NCT03356665. Prior to inclusion, each potential study participant was informed about the objectives, conduct, benefits and risks of the study in the language of their choice in order to obtain written informed consent. For children, parents/legal guardians provided written informed consent agreeing to their children’s participation in the study. In addition, assent was obtained from the participating minors.

#### Study area and inclusion and exclusion criteria

This study took place in 10 fixed health centers, including two centers for diagnosis and treatment (CDT) and eight serological screening site (SSS) in the Bonon and Sinfra endemic foci in west-central Côte d’Ivoire, as previously described [29]. From August 2017 to December 2019, patients who visited one of the selected fixed health centers were included in the study based on the following inclusion criteria: visitors or residents in a HAT endemic area and clinical suspicion of HAT based on one or more of the following clinical symptoms or signs: 1) long-term fever, resistant to antimalarial treatment, 2) headache for a long period (> 14 days), 3) presence of enlarged lymph nodes in the neck, 4) severe weight loss, 5) weakness 6) severe pruritus, 7) amenorrhea, abortion(s), or sterility, 8) psychiatric problems (aggressiveness, apathy, mental confusion, unusual increasing hilarity), 9) sleep disturbances (nocturnal insomnia and excessive daytime sleep), 10) motor disorders (abnormal movements, tremor, difficulty walking), 11) speech disorders, 12) convulsion, 13) coma. Individuals meeting any of the following criteria were excluded from the study: subjects previously treated for HAT; subjects refusing to sign the informed consent form; children <4 years.

#### Diagnostic procedure and sample collection

**Fig 1:**
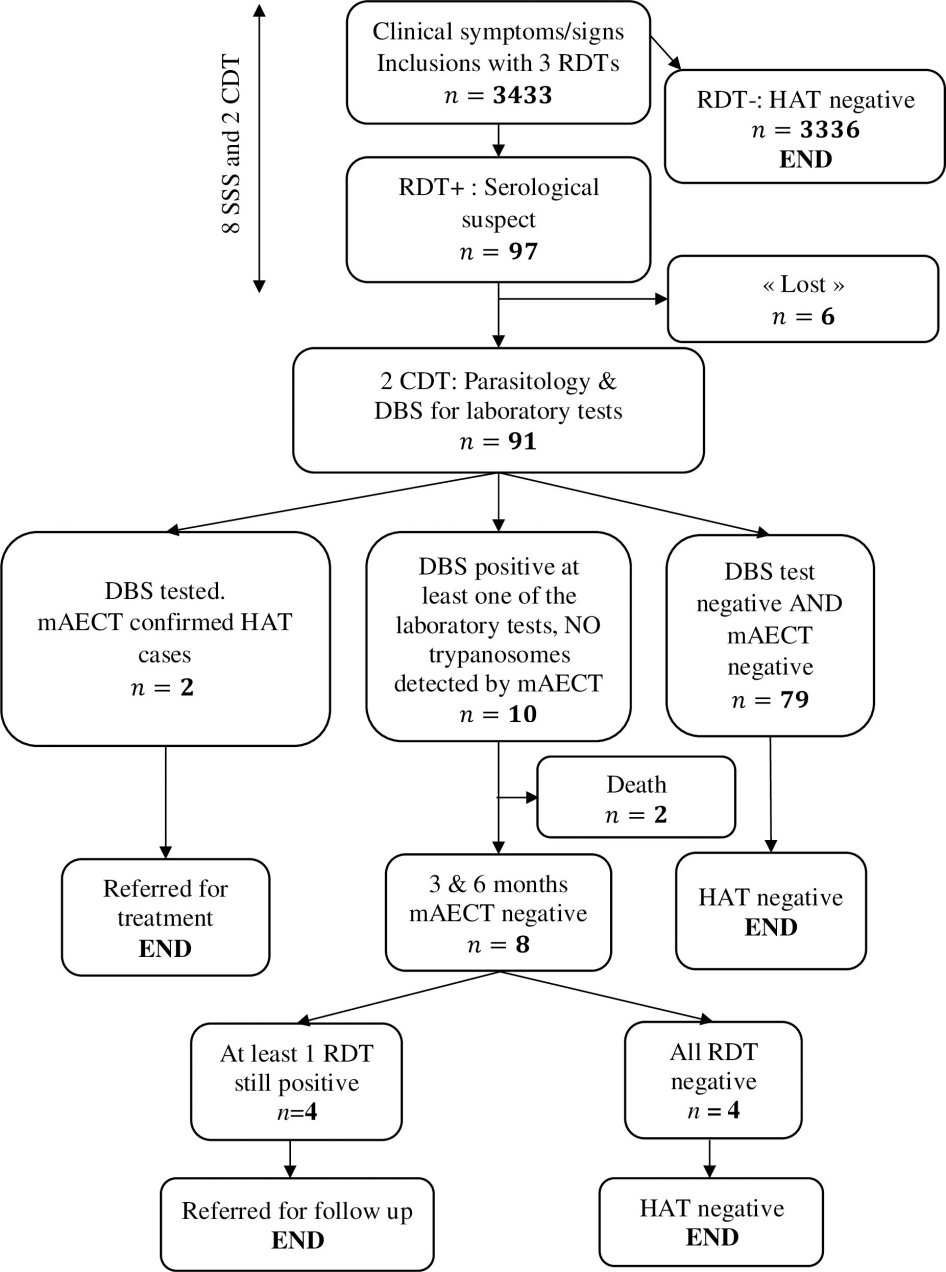
Flow chart of study participants with tests results. RDT rapid diagnostic test, HAT human African trypanosomiasis, SSS serological screening site, CDT centre for diagnosis and treatment, DBS dried blood spot, mAECT mini anion exchange centrifugation technique.

The diagnostic procedure is summarized in Fig 1. Following informed consent, 3 RDTs were performed from a blood sample taken by finger prick and using the blood transfer device of each test: SD Bioline HAT (Abbott Diagnostics, South Korea), HAT Sero-*K*-Set (Coris BioConcept, Belgium), and rHAT Sero-Strip (Coris BioConcept, Belgium). Tests were carried out in parallel and according to the manufacturer’s instructions. Subjects negative to the 3 RDTs were considered free from HAT. Subjects that tested positive for at least one RDT were considered “serological suspects” and were referred for parasitological examinations at the CDT. From each serological suspect, venous blood was taken using heparin as anti-coagulant, to carry out the mini-anion exchange centrifugation technique (mAECT) [30] according to the manufacturer’s instructions (INRB, Democratic Republic of Congo). If enlarged cervical lymph nodes were present, they were punctured to obtain fresh lymph, which was examined microscopically by direct examination at 10x40 magnification. In case of strong clinical suspicion, the CDT clinician could decide to perform a lumbar puncture for trypanosome detection in the cerebrospinal fluid by modified simple centrifugation [31]. HAT cases in whom trypanosomes were detected, were treated at the CDT according to the Ivorian national procedure implemented by the National Program for the Elimination of HAT (NPEHAT).

For every serological suspect two categories of dried blood spots (DBS) were prepared. For RT-qPCR, ELISA/*Tbg* and trypanolysis, a total of 16 individual drops, 30μL each, of heparinised blood were carefully deposited on a Whatman No 4 paper mounted on a drying rod. For LAMP, 180 μL of heparinised blood were mixed with 20 μL of 5% SDS solution (Sigma Aldrich) in an eppendorf tube. After 5 minutes of lysis, two drops of 40 μL of the mixture were deposited on Whatman No 1001 filter paper. The filter papers were left to dry, then placed in separate envelopes, which were inserted in hermetically sealed plastic bags containing silica gel.

The DBS were sent for analysis to the Centre International de Recherche-Développement sur l’Elevage en zone Subhumide (CIRDES, Bobo-Dioulasso, Burkina Faso). Antibodies were assessed with trypanolysis and ELISA/*Tbg*. The trypanolysis was carried out with 2 *Tbg* variable antigen types (LiTat 1.3 and LiTat 1.5) as previously described [16]. Trypanolysis was considered positive if at least one trypanosome variable antigen type was lysed for more than 50 percent. ELISA/*Tbg* was performed as previously described [19] with the following small modifications. Briefly, 6 mm confettis were punched out of the Whatman No 4 dried blood spots, and eluted with 720 μL of PBS-Blotto-Tween buffer overnight at 4°C. The specimen was tested against a mixture of purified *Tbg* LiTat 1.3 and LiTat 1.5 variable surface glycoproteins, coated each at a concentration of 1μg/ml. The test result was considered positive when the percentage positivity of the test specimen was higher than 30, i.e. when the optical density (O.D.) obtained with a test sample was above 30% of the O.D. obtained with the positive control included in the plate. Molecular tests on DBS included LAMP (Eiken Chemical, Taito-ku, Tokyo, Japan), m18S-qPCR and, if the latter was positive, TgsGp-qPCR [25].

If the results of all four laboratory tests on DBS were negative, the serological suspect was considered free from HAT and no further follow-up parasitological examinations were performed. In the event that at least one of the four laboratory tests was positive, two visits, 3 months and 6 months after the date of inclusion, were carried out in order to repeat the parasitological examinations. At the last visit, the RDTs that were positive at inclusion were repeated. Serological suspects that had remained parasitologically negative and remained reactive in at least one RDT, were referred to the NPEHAT for further follow-up.

For each study participant, socio-demographic characteristics (residence, age, gender, contacts, date of visit, travel details) and details of inclusion symptoms were collected using an electronic questionnaire and stored in an electronic database. In the CDTs, pictures were taken from the RDT result, and if present, a short video of the trypanosome was recorded [32].

### Statistical analysis

Statistical analyses were performed using R software (version 4.0.2, R Foundation for Statistical computing, Vienna, Austria) via RStudio (Version 1.1.456–2009–2018 RStudio, Inc., Boston, MY). Descriptive statistics were carried out to summarize the characteristics of participants. Specifically, quantitative data were expressed as a median with range, while categorical variables were expressed as a frequency with percentage. The prevalence of HAT in the study population was calculated based on those testing positive on RDTs with parasitological confirmation in lymph, blood or cerebrospinal fluid.

Logistic regression analysis using the lme4 package in R was performed to identify clinical symptoms and signs associated with overall RDT positivity, and with positivity of each RDT separately. First, univariable analyses were carried out to screen variables based on their unconditional association with the four separate outcomes (overall RDT positivity; SD Bioline HAT positivity; HAT Sero-*K*-Set; rHAT Sero-Strip). Next, multivariable logistic regression models were built for each outcome using variables that were marginally significant (p-value <0.1) in the univariable analysis, together with their 2-way interaction terms. Stepwise model selection was performed, and the model with the lowest Akaike information criterion (AIC) value was chosen as the final model for each outcome.

The results of the diagnostic tests were analysed to establish associations between all possible paired combinations of tests. Cohen’s Kappa (K) coefficient [33] with its confidence intervals was used as a measure of agreement between each pair of tests, and the level of agreement was expressed in terms of positive and negative agreement indices [34], representing respectively the proportion of agreement observed for positive and negative test results using irr package in R [35]. The interpretation of K was made according to Landis and Koch [36] (very bad agreement if K <0.00; bad if 0.00 ≤ K ≤ 0.20; moderate if 0.21 ≤ K ≤ 0.60; good if 0.61 ≤ K ≤0.80; excellent if 0.81 ≤ K ≤1.00). The test specificity was estimated, after exclusion from the dataset of RDT-positive subjects that were lost to follow-up, as the number of test negatives in the non-HAT test population, over the total non-HAT test population. The non-HAT test population used as a denominator consisted of all subjects in whom no parasites were detected (including subjects that tested negative to the RDTs and were therefore not examined in parasitology). The PPV of each individual test, and combination of tests in series or parallel, was calculated. The degree of significance adopted was 5%. The confidence intervals (CI) were all calculated at 95%.

## Results

### Description of recruited patients and results of the diagnostic algorithm

Between August 2017 and December 2019, 3433 clinical suspects were screened with the three RDTs. In total, 1,606 (46.8%) inclusions were made in the Bonon focus and 1,827 (53.2%) in the Sinfra focus. The gender ratio of the study participants was 0.96 with 1,681 men and 1,752 women. Their median age was 37 years (IQR: 26–50). The clinical symptoms and signs are described in Table 1, with weakness, headache and fever being most common.

**Table 1:** Frequency of clinical symptoms and signs and their association with RDT positivity.

Clinical symptoms and signs	% frequency (number)	Univariable *p-value*	Multivariable OR (CI)	Multivariable *p-value*
Weakness	57.1 (1960)	0.897		
Headache (> 14 days)	50.4 (1730)	0.521		
Long-term fever	48.9 (1678)	0.220		
Sleep disturbances	26.2 (900)	< 0.001***	2.8 (1.9–4.3)	1^−06^***
Severe weight loss	16 (550)	0.001**	1.7 (1.06–2.75)	0.02*
Severe pruritus	8.2 (283)	0.262		
Motor disorders	5.8 (200)	< 0.001***	2.7 (1.4–4.8)	0.002**
Amenorrhea, abortion(s), sterility	4.8 (165)	0.08^a^		
Enlarged lymph nodes	3.6 (123)	0.771		
Psychiatric problems	2.5 (85)	< 0.001***	2.5 (0.97–95)	0.04*
Speech disorders	0.96 (33)	< 0.001***		
Convulsions	0.87 (30)	< 0.001***	4.6 (1.5–14.0)	0.02*
Coma	0.3 (11)	0.209		
Intercept			0.015 (0.01–0.02)	< 2^−16^ ***

OR: odds ratio; CI: confidence interval

‘***’: high significance

‘**’: moderate significance

‘*’: slight significance

^a^: analysis carried out only on women

The flow chart in Fig 1 shows the results of the screening and laboratory tests. A total of 97 clinical suspects (2.83%, CI: 2.33–3.43) were positive on at least one RDT (59 detected at a SSS and 38 at a CDT). There was no significant difference in the proportion of serological suspects between Bonon and Sinfra foci (*p* = 0.588). Among the 59 serological suspects that were detected at one of the fixed health centers, six did not go to the CDT for the parasitological confirmation and DBS collection. The cervical lymph nodes present in 4 clinical suspects were small and could not be punctured. The 91 serological suspects were tested with mAECT and trypanosomes were detected in two individuals.

Of the 91 DBS of serological suspects sent to the reference laboratory, 12 samples were positive for at least one of the laboratory tests (details discussed below), including the two confirmed cases of HAT. Two serological suspects with positive laboratory test results died. The remaining eight serological suspects were parasitologically re-examined twice, but no cases were parasitologically positive at these follow-ups. At the second follow-up visit, four of the serological suspects were still positive in an RDT and they were sent to the NPEHAT for follow-up. The remaining four had become negative to all the RDTs and were declared free of HAT. The prevalence of confirmed HAT in the participating clinical suspects was therefore 0.06% (CI 0.02–0.2%).

### Detailed test results for 12 seropositive, laboratory test positive participants

Detailed results are shown in Table 2. The two HAT patients were adolescents, a girl (11 years old) and a boy (13 years old). The girl presented with terminal HAT and clinical symptoms and signs included weight loss, weakness, psychiatric problems, sleep disturbances, motor disorders, convulsions, and speech disorders. Her physical condition and diagnostic pathway are described in more detail elsewhere [29]. The boy presented with fever and sleep disturbances. Both HAT patients tested positive in all 3 RDTs, and trypanosomes were demonstrated in their blood by mAECT. After analysis of their CSF, they were both classified as stage 2 *Tbg* patients since trypanosomes were found in the girl’s CSF with a cytorachia of 160 cells/mm^3^, while a cytorachia of 240 cells/mm^3^ was observed for the boy. They were treated with nifurtimox eflornithine combination therapy as recommended by the national procedure. Both HAT patients were ELISA/*Tbg*, trypanolysis, and LAMP positive. The boy was positive in m18S-qPCR, but TgsGP-qPCR negative.

**Table 2:** Detailed test results and outcome of 12 RDT positive, laboratory test positive study participants.

Status	Gender/age	HAT Sero-*K*-Set	rHAT Sero-Strip	SD Bioline HAT	Trypanolysis LiTAT 1.3	Trypanolysis LiTAT 1.5	ELISA/*Tbg*	LAMP	m18S-qPCR	TgsGP-qPCR
HAT	M/13	+	+	+	+	+	+	+	+	-
HAT	F/11	+	+	+	+	-	+	+	-	ND
Death	M/28	+	+	-	+	+	-	+	+	-
Death	F/20	+	+	+	+	+	-	-	-	ND
Seropositive, DBS+	M/27	+ (+)	-	-	-	-	-	-	+	-
Seropositive, DBS+	M/53	+ (+)	-	-	+	+	+	-	-	ND
Seropositive, DBS+	F/36	+ (+)	-	+ (-)	-	-	-	-	+	-
Seropositive, DBS+	M/71	+ (+)	+ (-)	+ (-)	-	-	-	-	+	-
Seroconverter, DBS+	F/52	+ (-)	-	-	-	+	-	-	+	-
Seroconverter, DBS+	F/6	+ (-)	-	+ (-)	-	-	-	-	+	-
Seroconverter, DBS+	F/35	+ (-)	-	-	-	-	-	-	+	-
Seroconverter, DBS+	M/38	+ (-)	-	-	+	-	-	-	-	ND

HAT: human African trypanosomiasis; DBS: dried blood spot; M: male; F: female; ND: not done; (): RDT result after 6 months; +: positive; -: negative

The two deceased participants were a woman and a man aged 20 and 28 respectively; both had fever, headache, weight loss, weakness, sleep disturbances, motor disorders, and speech disorders. In addition, the man suffered from pruritus, the women from psychiatric problems and convulsions. The man was positive for HAT Sero-*K*-Set and rHAT Sero-Strip, but remained negative in mAECT at inclusion. He died a few days later. His DBS turned out to be positive in trypanolysis, LAMP and m18S-qPCR. The woman was positive for all RDTs but was mAECT negative. As she was trypanolysis positive, she was requested to attend a follow-up visit 3 months later, on which mAECT remained negative. Taking into account her clinical presentation, lumbar puncture was performed but CSF was normal. The woman refused the next follow-up visit, and eventually died.

The remaining eight individuals presented with fever (n = 4), sleep disturbance (n = 4), headache (n = 3), weakness (n = 3), weight loss (n = 1), motor disorders (n = 1), and convulsion (n = 1). All eight were positive in HAT Sero-*K*-Set, three in SD Bioline HAT, and one in rHAT Sero-Strip. They were all mAECT negative at inclusion. For 6 seropositives the DBS was positive for m18S-qPCR but negative for TgsGP-qPCR, while trypanolysis and ELISA/*Tbg* were positive in respectively 3 and one individual. No-one was LAMP positive. All 8 remained mAECT negative at the 3- and 6-month follow-up examinations. At the 6 month follow-up visit, however, 4 individuals had seroconverted in the initially positive RDT(s), while 4 others maintained their HAT Sero-*K*-Set positivity.

### Clinical symptoms and signs as a predictor of being a serological suspect

Neither age (*p* = 0.149), nor gender (*p* = 0.918) were associated with being a serological suspect. Univariable analysis of clinical symptoms and signs in the study population showed that 6 out of 13 inclusion symptoms and signs (sleep disturbances, severe weight loss, motor disorders, psychiatric problems, convulsions and speech disorders) were significantly associated with positivity for at least one of the RDTs (Table 1). These six clinical symptoms and signs were therefore included in the multivariable regression model.

Stepwise model selection (Table 1), indicated that the best fitting and most parsimonious model (AIC = 841.86) was the one containing five clinical symptoms and signs: sleep disturbances, motor disorders, convulsions, severe weight loss, and psychiatric problems. The highest odds ratio was for convulsions, with the model indicating that the odds of a clinical suspect with convulsions testing positive on at least one RDT was 4.6 times higher (1.5–14.0, *p* = 0.02) than the odds of a clinical suspect without convulsions.

Univariable analysis for the three RDTs separately indicated that positivity to one of the RDTs was statistically significantly (p<0.001) associated with positivity to another RDT (S1 Table). The number of clinical symptoms and signs significantly associated with positivity to each RDT ranged between three and five, and these were the same identified as being associated with overall RDT positivity; sleep disorders and convulsions were significantly associated with positivity to each of the three RDTs (S2 Table).

### Performance of diagnostic tests and agreement between tests

When multiple diagnostic tests are available, combinations of tests can be interpreted in parallel (an individual who is positive on any one of the tests is considered a positive result) or in series (all tests must be positive for an individual to be considered positive). As indicated in Table 3, the HAT Sero-*K*-Set test reported the highest positivity rate (2.5%, CI: 2.0–3.1%) among the 3 RDTs. Samples testing positive with the m18S-qPCR were all negative for TgsGp-qPCR.

**Table 3:** Performance of the different rapid diagnostic tests for HAT using the parasitological test as gold standard.

Diagnostic test	Total positive	Positivity rate (95% CI)	Specificity (95% CI)^a^^,^^b^	PPV (95% CI)^b^
SD Bioline HAT	43	1.3 (0.9–1.7)	98.9 (98.5–99.2)	4.9 (1–17)
HAT Sero-*K*-Set	85	2.5 (2.0–3.1)	97.8 (97.2–98.2)	2.5 (0–9)
rHAT Sero-Strip	14	0.4 (0.2–0.7)	99.6 (99.4–99.8)	14.3 (2–43)
All RDTs (in parallel)	97	2.8 (2.3–3.4)	97.2 (96.6–97.7)	2.2 (0–8)
All RDTs (in series)	6	0.2 (0.1–0.4)	99.9 (99.7–100)	33.3 (4–78)
HAT Sero*-K-*Set- rHAT Sero-Strip (parallel)	85	2.5 (2.0–3.1)	97.8 (97.2–98.2)	2.5 (0–9)
HAT Sero-*K*-Set- SD Bioline HAT (parallel)	97	2.8 (2.3–3.4)	97.2 (96.6–97.8)	2.2 (0–8)
rHAT Sero Strip- SD Bioline HAT (parallel)	51	1.5 (1.2–2.0)	98.6 (98.2–99.0)	4.1 (0–14)
HAT Sero-*K*-set + rHAT Sero Strip (series)	14	0.4 (0.2–0.7)	99.6 (99.4–99.8)	14.3 (2–43)
HAT Sero-*K*-set + SD Bioline (series)	31	0.9 (0.6–1.3)	99.2 (98.9–99.5)	6.9 (1–23)
rHAT Sero Strip + SD Bioline HAT (series)	6	0.2 (0.1–0.4)	99.9 (99.7–100)	33.3 (4–78)

^a^: denominator of 3425 for RDTs

^b^: 6 lost individuals excluded for these calculations (4 positive in HAT Sero-*K*-Set only, and 2 positive in HAT Sero-*K*-Set + SD Bioline HAT)

The specificities of the RDTs ranged from 97.2% (parallel combination) to 99.9% (serial combination). For these calculations, the 6 lost individuals were excluded (4 positive in HAT Sero-*K*-Set only, and 2 positive in HAT Sero-*K*-Set + SD Bioline HAT). Of the 3 RDTs, rHAT Sero-Strip was the most specific with 99.6% (3413/3425, CI 99.4–99.8%). The cassette-based RDTs were slightly less specific, with the SD Bioline HAT (98.9%; CI 98.5–99.2%) being more specific than the HAT Sero-*K*-Set (97.8%; CI 97.2–98.2%). The series combinations of all the RDTs and rHAT Sero-Strip + SD Bioline HAT had the highest specificity (3421/3425, 99.9%; CI: 99.7–100%). Among the laboratory tests performed on the 91 RDT seropositives (Table 4), specificities ranged from 93.3% to 98.9%, disregarding TgsGp-qPCR. LAMP and ELISA/*Tbg* had the highest specificity of 98.9% (88/89, CI: 93.9–99.9%). Sensitivity was not calculated.

**Table 4:** Performance of laboratory tests performed on dried blood spots of RDT seropositive subjects for diagnosis of HAT, using the parasitological test as gold standard.

Diagnostic test	Total positive	Positivity rate (95% CI)	Specificity (95% CI)^a^	PPV (95% CI)
Trypanolysis	7	7.7 (3.8–15)	94.4 (87.4–98.1)	29 (4–71)
LAMP	3	3.3 (1.1–9.3)	98.9 (93.9–99.9)	67 (9–99)
ELISA/*Tbg*	3	3.3 (1.1–9.3)	98.9 (93.9–99.9)	67 (9–99)
m18s-qPCR	8	8.8 (4.5–16.4)	93.3 (85.7–97.5)	25 (4–89)
TgsGp-qPCR	0	0	1 (0.96–1)	---

^a^: denominator of 89 (except for TgsGp-qPCR which had a denominator of 8)

The PPV of the tests was evaluated as the ability to predict HAT (Table 3). The estimated PPV for the RDTs was generally low, with the highest PPV being 33% (2/6, 95% CI 4–78%) for both the ‘in series’ interpretation of all three RDTs and the ‘in series’ interpretation of rHAT Sero-Strip and SD Bioline HAT. The ‘in-series’ addition of the HAT Sero-*K*-Set did not improve the PPV. The laboratory tests, performed on RDT pre-screened samples, had PPVs ranging from 25 (2/8, CI 4–89%) for m18s-qPCR, to 67% (2/3, CI 9–99%) for LAMP and ELISA/*Tbg* (Table 4).

Table 5 details the agreement between tests. The kappa coefficient for the RDTs was calculated on 3433 RDT results. Agreement between the 3 RDTs was moderate for each 2-way comparison. Kappa coefficients were calculated between the laboratory tests and between laboratory and RDT results on the 91 available results. Agreement between the RDTs and the laboratory tests showed an overall poor agreement, except for trypanolysis and rHAT Sero-Strip which had moderate agreement (K = 0.3; CI 0.02–0.6). Laboratory tests showed moderate agreement, except for ELISA/*Tbg* and LAMP tests which had a good level of agreement (K = 0.7; CI: 0.2–1.1).

**Table 5:** Agreement between HAT Sero-*K*-Set, rHAT Sero-Strip, SD Bioline HAT, trypanolysis, ELISA/*Tbg* and LAMP.

	SD Bioline HAT		rHAT Sero-Strip		Trypanolysis		ELISA/*Tbg*		m18S-qPCR		LAMP	
	Pos	Neg	Pos	Neg	Pos	Neg	Pos	Neg	Pos	Neg	Pos	Neg
HAT Sero-*K*-Set												
Pos	31	54	15	70	7	72	3	76	8	71	3	76
Neg	12	3336	0	3348	0	12	0	12	0	12	0	12
Kappa (CI)	**0.47** (0.36; 0.58)		**0.27** (0.16; 0.39)		**0.02** (0.00; 0.05)		**0.01** (-0.03; 0.02)		**0.03** (0.00; 0.05)		**0.01** (-0.03; 0.02)	
SD Bioline HAT												
Pos			7	36	4	36	2	38	4	36	2	38
Neg			8	3382	3	48	1	50	4	47	1	50
Kappa (CI)			**0.20** (0.06; 0.30)		**0.04** (-0.08; 0.17)		**0.03** (-0.05; 0.12)		**0.02** (-0.11; 0.15)		**0.03** (-0.05; 0.12)	
rHAT Sero-Strip												
Pos					4	11	2	13	3	12	3	12
Neg					3	73	1	75	5	71	0	76
Kappa (CI)					**0.29** (0.02; 0.56)		**0.18** (-0.06; 0.42)		**0.16** (-0.08; 0.41)		**0.29** (0.03; 0.56)	
Trypanolysis												
Pos							3	4	3	4	3	4
Neg							0	84	5	79	0	84
Kappa (CI)							**0.58** (0.22; 0.95)		**0.35** (0.02; 0.68)		**0.58** (0.22; 0.95)	
ELISA/*Tbg*												
Pos									1	3	2	1
Neg									7	81	1	87
Kappa (CI)									**0.14** (-0.17; 0.45)		**0.66** (0.21; 1.10)	
m18S-qPCR												
Pos											2	6
Neg											1	82
Kappa (CI)											**0.33** (-0.03; 0.70)	

Kappa values are in bold, CI: confidence interval; Pos: positive; Neg: negative

## Discussion

The low *Tbg* HAT prevalence observed in passive screening in Central-West in Côte d’Ivoire can be considered a strength. Sporadic HAT occurrence was expected: the annual number of HAT cases reported since 2009 has always been below 10 [37], and low prevalence were reported in previous studies [38,39]. Côte d’Ivoire presents characteristics of having eliminated HAT as a public health problem [5], evolving towards zero transmission. Côte d’Ivoire was therefore considered as a model for the West-African region where adapted diagnostic algorithms for passive case detection and disease surveillance are highly relevant. Previous prospective RDT evaluation studies however have all been carried out in Central Africa [11,12,14,40]. The 0.06% prevalence we observed contrasts with the 1.13% prevalence observed in passive case detection in a previous phase 3 study conducted in Democratic Republic of the Congo (DR Congo) [11]. However, with only two HAT cases confirmed in the present study, extreme care should be taken with interpretation of diagnostic test sensitivity, which was therefore not calculated; this can be considered as a limitation of the study.

The most important strength of the present study is the prospective parallel evaluation, for the first time, of all available RDTs for diagnosis of HAT. Previous prospective studies evaluated either RDTs produced by Standard Diagnostics [11,12], or by Coris BioConcept [14,40]. Only one study compared RDTs of both test producers on stored plasma originating from West-Africa [41]. An additional strength is the parallel examination of DBS in 4 different laboratory tests: trypanolysis, ELISA/*Tbg*, LAMP and qPCR. Dried blood spot testing of seropositive unconfirmed subjects to select individuals for additional parasitological follow-up has previously been performed either with serology, in trypanolysis and/or ELISA/*Tbg* [16,19], or with molecular tests [12]. So far, LAMP has been mainly applied in the field on seropositive microscopy negative subjects, to compensate for the imperfect sensitivity of microscopy [26,42]. Serological and molecular tests have rarely been performed in parallel [24].

Unfortunately, the study set-up has some inherent weaknesses. First, individuals without one of the 13 clinical inclusion symptoms and signs for HAT were not included. Some true HAT patients might have been missed, in particular first stage patients with mild symptoms. This limitation is inherent to the passive case detection setting. In the present study, we indeed only picked up patients with severe late stage HAT, which were probably infected long ago.

On the other hand, we cannot guarantee that all individuals presenting at the health centre with one of the 13 clinical symptoms and signs for inclusion were effectively included. Although we tried to sensitize the majority of the health staff working in the study sites, HAT is perceived as a disease from the past in Côte d’Ivoire, and many health professionals are unaware of it, even in endemic foci [29]. Furthermore, we assumed that clinical suspects negative in all 3 RDTs did not suffer from HAT, so they were not examined neither parasitologically nor in the four laboratory tests. Although sensitivity of the individual RDTs might be lower than 100%, we assumed that a combination of 3 RDTs would detect different serological profiles, and bring sensitivity close to 100%. This approach might result in an overestimation of the specificity of the RDTs, as triple RDT negative HAT cases were considered non-HAT, but taking into account the very low HAT prevalence in Côte d’Ivoire, the effect is likely to be small. Previous prospective trials [11,12] also did not perform parasitology systematically on all RDT or CATT negatives. Reasons are the strong increase in costs, infrastructural requirements (8/10 health centres were not equipped to do parasitology), and work load, which was already experienced to be high by the health personnel due to administrative and ethical aspects of the diagnostic trial. A similar limitation is that we considered participants who tested negative in all laboratory tests as non-HAT, and such subjects were therefore classified as false RDT positives.

Two other serological screening tests for HAT were not performed in this study. A promising additional RDT, SD Bioline HAT 2.0, based on recombinant LiTat 1.5 VSG and ISG 65 antigens, would be more sensitive than SD Bioline HAT [11]. Unfortunately, the test remained unavailable for the whole study duration. Although parallel screening with CATT might theoretically have been interesting, also for comparison with previous diagnostic studies [11,12,14,40], it was considered irrelevant for passive screening. Indeed, with 3433 participants in 10 centres over 29 months, on average 12 tests/centre/month were performed, which would have implied considerable waste of CATT reagent, in particular in centres not able to store the reagent in the fridge. Attendance rates and test use of health centres offering HAT diagnosis in some HAT endemic areas can be extremely low [43].

The imperfect sensitivity of mAECT implies that parasitological confirmation in some RDT true positives might have been missed. However, RDT positives that were positive in the laboratory tests were followed up with two additional parasitological examinations wherever possible. We therefore made a strong effort to confirm RDT positives. Unfortunately, two seropositive laboratory test positive participants died without receiving the complete parasitological follow-up. They might have been HAT patients. As previously described, different mAECT examinations might be necessary for confirmation [29]. For the same reason, study participants who were followed up and remained RDT positive until the end of the study might have been real cases, and were therefore referred to the NEPHAT for follow-up.

The present study obtained a high referral rate of RDT seropositives to parasitology. Indeed, 53/59 (89.8%) of RDT seropositives detected at the fixed health centres (SSS) where parasitological examination was not available, attended the onwards parasitological confirmation step at the level of the CDT. This is slightly more than proportions reported elsewhere [26]. In passive screening in Kongo Central in DR Congo however, only 39.9% of RDT positives referred for confirmatory testing to the CDT completed their referral [42]. Although the latter authors do not exclude that the group that did not complete the diagnostic examinations might have contained HAT cases, they considered it probable that most lost seropositives would have had a self-limiting illness and recovered. The 6 “lost” seropositives had relatively mild symptoms (mainly weakness, headache and fever, sleep disturbance for one), and tested positive in one or maximally two RDTs. Poor communication by the health personnel about HAT RDTs also has been highlighted as a reason for non-completion [43]. In the present study in Côte d’Ivoire, regular meetings with the country study coordinators might have improved understanding and motivation of the health personnel, resulting in better efforts in communication towards study participants. Active tracing of RDT positives not showing up for confirmation parasitology was carried out, and they were encouraged to go to the CDT.

Selection of 13 inclusion symptoms and signs for the actual study was based on the WHO guidelines for passive surveillance. For all study participants, presence of these symptoms and signs was systematically recorded. Although the frequency of clinical symptoms and signs in *Tbg* HAT patients has been described previously [44–48], clinical criteria with increased odds for a positive RDT or for HAT in a population visiting the hospital have been rarely studied. Application of simple clinical algorithms facilitates selection of individuals to be tested for HAT. Such an algorithm to select individuals for RDT testing, could in Côte d’Ivoire be based on presence of sleep disturbances, motor disorders, convulsions, severe weight loss or psychiatric problems. The absence of enlarged cervical lymph nodes, in particular in all 12 individuals with HAT or with high suspicion of HAT, was remarkable since it is considered typical for HAT and was the most frequent sign observed in 86.3% of HAT patients in the neighbouring focus of Daloa [44]. In South Sudan, syndromic referral algorithms consisting of sleep problems, neurological problems, weight loss and/or a history of oedema would best identify HAT among a treatment-seeking population [49]. Whether the same clinical criteria to select RDT seropositives would be appropriate in other HAT endemic countries remains to be confirmed.

Our specificity estimates of 98.9% for SD Bioline HAT and 97.8% for HAT Sero-*K*-Set in Côte d’Ivoire approach specificities previously reported for those tests in prospective trials in Central Africa. For SD Bioline HAT, specificities of 94.6% and 98.8% have been reported previously [11,12], while the latter also reports a slightly lower 96.7% specificity value in passive case detection. Specificity values reported previously from DR Congo for HAT Sero-*K*-Set were 98.6 and 97.0% [14,40]. Specificities of around 88%, observed with SD Bioline HAT and HAT Sero-*K*-Set using stored plasma from West-Africa [41], were lower. For the laboratory tests, specificities reported previously in DR Congo were 99.8% for ELISA/*Tbg* on DBS [19], and 98.0% for trypanolysis on serum [14]. The specificity of ELISA/*Tbg* and trypanolysis in the present study, of respectively 98.9% and 94.4%, are probably underestimated, as these tests were carried out on RDT positives only, which partially rely on the same antigens, *Tbg* LiTat 1.3 and 1.5 VSGs, for antibody detection. This does not apply to the molecular tests, for which the 98.9% specificity observed in the present study compares well with LAMP specificities of 92.8% and 96.4% observed in a laboratory study on extracted DNA [50].

The PPV and agreement of most tests was relatively low, compared to previous observations, driven strongly by the underlying low prevalence in the Bonon and Sinfra foci. In DR Congo, the PPV of SD Bioline HAT in passive case detection was 14.4% [42], while among patients with neurological disorders, the PPV of HAT Sero-K-Set was 50% [40], for HAT prevalences of respectively 0.1–0.8% and 2.9%. We observed PPVs for these RDTs of 4.9% and 2.5%, and 14.3% for rHAT Sero-Strip. For the laboratory tests, PPVs were between 29 and 67%, but these tests were performed on a RDT positive population, therefore pre-selecting a higher prevalence population (2 out of 91 seropositives or 2.4%). As a result of the elevated specificity of the tests in combination with the low HAT prevalence, the confidence intervals from the PPV were however wide. Similarly, the agreement between RDTs was rather low. The sensitivity of the kappa coefficient to prevalence results in an "abnormally low" Kappa value [51]. Also the agreement between the RDTs and the laboratory tests ranged from low to moderate, which might be due to the fact that not all the tests are based on the same biological principle. Good agreement was found between two laboratory tests (ELISA/*Tbg* and LAMP), suggesting that these two techniques have more consistent results and that potentially only one of the two tests could be used in a future diagnostic algorithm.

Four subjects were RDT positive, laboratory tests positive and mAECT negative, but sero-converted to RDT negative by the end of the trial. At the end of the study, however, 4 subjects remained RDT positive, laboratory test positive, yet were parasitically unconfirmed after 3 mAECT tests. In addition 2 subjects died, and we cannot confirm nor exclude that they had HAT. The four remaining RDT positive individuals remain under continued follow-up to exclude the risk that they would act as aparasitemic serological suspects becoming asymptomatic carriers [52–54], who may act as a human parasite reservoir and can maintain transmission. We cannot exclude that RDT positive laboratory test positive individuals might carry trypanosomes, including in the brain or skin [55].

Future treatment regimens with an improved safety profile may open up the possibility of treating such highly suspect cases, without parasitological confirmation [28]. The diagnostic criteria to identify these highly suspicious cases will be an important next step in the fight against HAT. However, test positivity could possibly also be explained by a transient infection with animal trypanosomes. Animal trypanosome infection rates of cattle and pigs in the foci of Bonon and Sinfra, where the present study took place, are quite high [56]. Infected tsetse flies may also bite humans and during the bite, inject animal trypanosomes, which might elicit an antibody response and false positivity in serological tests, or transient false positivity in molecular tests which are *Trypanozoon* specific, such as m18S-qPCR. The *Tbg* specific TgsGP-qPCR, which was carried out on all m18S-qPCR positive DBS, was always negative, including in the 2 confirmed HAT patients. With its limited sensitivity [25], this test does not seem to be helpful in detecting *Tbg* HAT.

The present study has some practical impacts for HAT control in Côte d’Ivoire. It has initiated the set-up of a passive case detection network in the country. This passive screening network is now being enlarged through other projects [57]. The intention is to maximally cover known HAT foci, and, taking into account the mobility of the population, also include key health structures in non-endemic districts which might be frequented by HAT cases seeking for care [29]. Furthermore, the study allowed passive screening of 3433 individuals for HAT in the last 2 HAT endemic districts in Côte d’Ivoire and contributed to the elaboration of a WHO dossier for validation of HAT elimination as a public health problem in Côte d’Ivoire. This underlines again that participation to clinical research can contribute to strengthening of health systems, as previously described for DR Congo [58].

Despite the limitations of the current study, some practical recommendations can be derived. The list of clinical symptoms and signs for selection of clinical suspects to be screened for HAT RDT testing in Côte d’Ivoire might be reduced to 5 key clinical signs, possibly extended with presence of enlarged cervical lymph nodes, which is known to be suggestive for HAT, and if confirmed in other project countries with higher HAT prevalences. The results also confirm the appropriateness of the diagnostic test algorithm that is presently applied in routine in Côte d’Ivoire. Serological screening can be performed with any of the three commercially available RDTs. For RDT positive subjects, the actual algorithm depends on on-site availability of parasitology. Seropositives are either directly examined in parasitology and if HAT is not confirmed, a DBS is taken for further testing with trypanolysis to decide on follow-up. Alternatively, if parasitology is not immediately available, a DBS can first be taken and tested in trypanolysis or ELISA/*Tbg*, two methods that were recently implemented at the national HAT diagnosis reference laboratory of Institut Pierre Richet, Bouaké. In that case, if the DBS is positive, specific actions can be initiated for the individuals to undergo parasitology.

Some open questions and topics for future research remain. The current study does not yet determine the optimal diagnostic algorithm for passive case detection in terms of sensitivity, specificity and cost-effectiveness. Further analysis of the study results, including results from other countries is therefore indicated. Combining results from different test populations will allow estimation of the diagnostic sensitivity and specificity in the absence of a gold standard using latent class analysis [59]. The next step is the cost-effectiveness analysis, allowing to choose the most appropriate HAT diagnostic test algorithm for the Ivorian context.

## Supporting information

S1 Table Univariable associations between the explanatory variables of interest and positivity with each of the three Rapid Diagnostic Tests used in this study. (DOCX)

S2 Table Multivariable logistic regression models for associations between clinical symptoms and signs and positivity with each of the three Rapid Diagnostic Tests. (DOCX)
