# Supplementary material for: Passive surveillance of human African trypanosomiasis in Côte d’Ivoire: Understanding prevalence, clinical symptoms and signs, and diagnostic test characteristics
Source: PLoS Negl Trop Dis. 2021 Aug 30;15(8):e0009656. doi: 10.1371/journal.pntd.0009656 (PMC8432893; doi:10.1371/journal.pntd.0009656)
Supplement: S1 Table — (DOCX) [file pntd.0009656.s001.docx]

| **Explanatory variable** | **Rapid Diagnostic Test** | | |
| --- | --- | --- | --- |
|  | **SD Bioline HAT positivity**  (n=43; 1.25%) | **HAT Sero-*K*-Set positivity**  (n=85; 2.48%) | **rHAT Sero-Strip positivity**  (n=14; 0.41%) |
| **Demographics** |  |  |  |
| Age | 0.380 | 0.040** | 0.855 |
| Foyer | 0.375 | 0.351 | 0.436 |
| Gender | 0.348 | 0.565 | 0.092* |
|  |  |  |  |
| **Clinical symptoms & signs** |  |  |  |
| Weakness | 0.631 | 0.917 | 0.997 |
| Headache (>14 days) | 0.837 | 0.256 | 0.976 |
| Long-term fever | 0.427 | 0.215 | 0.850 |
| Sleep disturbances | < 0.001*** | < 0.001*** | < 0.001*** |
| Severe weight loss | 0.085* | 0.012** | 0.200 |
| Severe pruritus | 0.389 | 0.423 | 0.881 |
| Motor disorders | 0.022** | < 0.001*** | < 0.001*** |
| Enlarged lymph nodes | 0.704 | 0.979 | 0.473 |
| Psychiatric problems | < 0.001*** | < 0.001*** | 0.004*** |
| Speech disorders | < 0.001*** | < 0.001*** | < 0.001*** |
| Convulsions | < 0.001*** | < 0.001*** | < 0.001*** |
| Coma | 0.019** | 0.597 | 0.832 |
|  |  |  |  |
| **Rapid diagnostic test** |  |  |  |
| SD Bioline HAT positivity | N/A | < 0.001*** | < 0.001*** |
| HAT Sero-K-Set positivity | < 0.001*** | N/A | < 0.001*** |
| rHAT Sero-Strip positivity | < 0.001*** | < 0.001*** | N/A |

**S1 Table. Univariable associations between the explanatory variables of interest and positivity with each of the three Rapid Diagnostic Tests used in this study**

*p-value<0.10; **p-value<0.05; ***p-value<0.001
