# Supplementary material for: Passive surveillance of human African trypanosomiasis in Côte d’Ivoire: Understanding prevalence, clinical symptoms and signs, and diagnostic test characteristics
Source: PLoS Negl Trop Dis. 2021 Aug 30;15(8):e0009656. doi: 10.1371/journal.pntd.0009656 (PMC8432893; doi:10.1371/journal.pntd.0009656)
Supplement: S2 Table — (DOCX) [file pntd.0009656.s002.docx]

**S2 Table. Multivariable logistic regression models for associations between clinical symptoms and signs and positivity with each of the three Rapid Diagnostic Tests**

| **Explanatory variable** | **Odds Ratio** | **95% Confidence Intervals** | **p-value** |
| --- | --- | --- | --- |
| **SD Bioline HAT** |  |  |  |
| Sleep disturbances | 4.06 | 2.18 - 7.74 | <0.001*** |
| Psychiatric problems | 3.62 | 1.07 - 9.67 | 0.02* |
| Convulsions | 14.25 | 3.51 - 45.88 | <0.001*** |
| Intercept | 0.01 | 0.003 - 0.01 | <0.001 *** |
|  |  |  |  |
| **HAT Sero-*K*-Set** |  |  |  |
| Age | 0.99 | 0.97 - 1.00 | 0.05* |
| Sleep disturbances | 2.89 | 1.84 4.52 | <0.001*** |
| Severe weight loss | 1.56 | 0.91 - 2.57 | 0.09* |
| Motor disorders | 2.89 | 1.47 - 5.29 | 0.001** |
| Psychiatric problems | 2.74 | 1.07 - 6.06 | 0.02** |
| Convulsions | 3.93 | 0.94 - 12.50 | 0.04** |
| Intercept | 0.02 | 0.01 - 0.04 | <0.001*** |
|  |  |  |  |
| **rHAT Sero-Strip** |  |  |  |
| Gender | 2.93 | 0.90 - 12.22 | 0.09* |
| Sleep disturbances | 12.21 | 3.52 - 61.71 | <0.001*** |
| Speech disorders | 14.27 | 2.05 - 67.36 | <0.001** |
| Convulsions | 18.88 | 1.65 - 166.05 | 0.01** |
| Intercept | 0.0004 | 0.000055 - 0.0016 | <0.001*** |

*p-value<0.10; **p-value<0.05; ***p-value<0.001
